# Corticotropin Releasing Hormone Signaling in the Bed Nuclei of the Stria Terminalis as a Link to Maladaptive Behaviors

**DOI:** 10.3389/fnins.2021.642379

**Published:** 2021-03-19

**Authors:** Claire Emily Young, Qingchun Tong

**Affiliations:** ^1^The Brown Foundation Institute of Molecular Medicine, The University of Texas Health Science Center at Houston, Houston, TX, United States; ^2^Department of Neurobiology and Anatomy of McGovern Medical School, The University of Texas Health Science Center at Houston, Houston, TX, United States; ^3^MD Anderson Cancer Center & UTHealth Graduate School of Biological Sciences, The University of Texas Health Science Center at Houston, Houston, TX, United States

**Keywords:** BNST, CRH, CRHR1, extended amygdala, anxiety, addiction

## Abstract

The bed nuclei of the stria terminalis (BST) is a limbic region in the extended amygdala that is heavily implicated in anxiety processing and hypothalamic-adrenal-pituitary (HPA) axis activation. The BST is complex, with many nuclei expressing different neurotransmitters and receptors involved in a variety of signaling pathways. One neurotransmitter that helps link its functions is corticotropin releasing hormone (CRH). BST CRH neuron activation may cause both anxiogenic and anxiolytic effects in rodents, and CRH neurons interact with other neuron types to influence anxiety-like responses as well as alcohol and drug–seeking behavior. This review covers the link between BST CRH neurons and thirteen other neurotransmitters and receptors and analyzes their effect on rodent behavior. Additionally, it covers the translational potential of targeting CRH signaling pathways for the treatment of human mental health disorders. Given the massive impact of anxiety, mood, and substance use disorders on our society, further research into BST CRH signaling is critical to alleviate the social and economic burdens of those disorders.

## Introduction

Approximately one in three people in the United States will develop an anxiety disorder during their lifetime (Craske et al., 2017). The bed nuclei of the stria terminalis (BST; also known as BNST) is a small, sexually dimorphic, heterogeneous region that in the past 20 years, has been highlighted for its role in stress and anxiety responses (for review, see Lebow and Chen, 2016). While traditionally BST research has focused on anxiety processing, in recent years the BST has been increasingly implicated in dysregulated signaling patterns consistent with many mental health disorders in both animal models and humans (Gungor and Paré, 2016; Lebow and Chen, 2016; Luyten et al., 2016; Blomstedt et al., 2017; Knight and Depue, 2019). Given the economic and social burden of those disorders on our society today, further study of the BST is a key step in the development of more effective treatments.

The BST circuitry is complex, involving neural connections both between the BST and other brain regions, and within the different nuclei of the BST. The BST connectivity has been discussed extensively elsewhere (see Bota et al., 2012; Vranjkovic et al., 2017; Ch’ng et al., 2018) and will not be elaborated upon in detail here. In brief, the BST is commonly grouped with the amygdala as part of the extended amygdala because of their proximity and similar cellular composition and immunohistochemistry (Alheid and Heimer, 1988). The two regions are also connected through signaling circuits like that of corticotropin releasing hormone (CRH; also known as CRF) between the central amygdala (CEA) and anteromedial BST (BSTam) (Pomrenze et al., 2019). More recent studies have demonstrated a role for the BST in anxiety processing independent of amygdalar inputs, and they underscore the need for more research specifically focusing on the BST.

Preclinical models are important for advancing the understanding of mental health disorders because they allow for the precise modification of animal brains. Previous research has demonstrated that the BST is involved in the processing of anxiety-like behavior in animal models, characterized by sustained fear due to conditioned or unpredictable cues (Walker and Davis, 1997; Walker et al., 2003; Daldrup et al., 2016), while the amygdala is more involved in the processing of phasic fear induced by short, discrete cues (Davis et al., 2010). The distinction that animals display anxiety-like behavior, and not anxiety, is necessary since their behavior is measured using metrics that are assumed to represent anxiety in animals, such as time spent in the center of an open field (OF) test (Carola et al., 2002), but cannot be confirmed to truly represent anxiety since anxiety as traditionally conceptualized is a subjective state (LeDoux and Pine, 2016). The term “anxiety-like” acknowledges that attributing behaviors to anxiety in animals is a form of anthropomorphism and may not be accurate.

An additional nuance to BST research is the distinction between anxiety and fear. The terms “fear” and “anxiety” are often used interchangeably, both in popular culture and by scientists and medical professionals, and Shackman and Fox (2016) argue that saying “fear and anxiety” together in reference to either will allow for less confusion about the term while focusing on the threat context. However, included in the threat context is the threat imminence, and other researchers such as Gungor and Paré (2016) argue that fear can be categorized as a defensive response to an imminent threat, while anxiety is a response to a diffuse, or not imminent threat. The majority of papers referenced in this review focus on what would be classically considered as anxiety-like responses. While early models created a sharp distinction between the CEA and BST in fear and anxiety processing, more recent research has demonstrated that both regions have a role in processing both uncertain and certain threat contexts, and thus may influence fear and anxiety processing (Shackman and Fox, 2016).

The BST is involved in the development of other maladaptive behaviors beyond anxiety disorders. It has been implicated in binge drinking and alcohol use disorders (AUDs), particularly the stress-induced reinstatement of drug-seeking behaviors (Silberman and Winder, 2013; Nentwig et al., 2019; Snyder et al., 2019). In this model of the addiction cycle, withdrawal produces negative affect and dysregulated BST CRH signaling. Stress can then induce the reinstatement of drug-seeking behavior (for review, see Silberman and Winder, 2013). The BST is also involved in the regulation of depressive-like behaviors, an effect that is CRH-dependent (Salimando et al., 2020). Depressive-like behavior is typically quantified in preclinical models by tests such as the forced swim (FS) and tail suspension tests that measure parameters like helplessness and anhedonia (Krishnan and Nestler, 2011). Finally, several results discussed in this review implicate BST circuits in the regulation of feeding behavior. In humans, feeding behavior is psychologically complex and not driven solely by the demands of energy metabolism, and thus may be difficult to investigate in preclinical models, particularly in relation to maladaptive behaviors (Ellacott et al., 2010). The link between the BST and maladaptive behaviors beyond anxiety is an emerging field of research, and CRH signaling within the BST presents new treatment targets for these behaviors.

**FIGURE 1:**
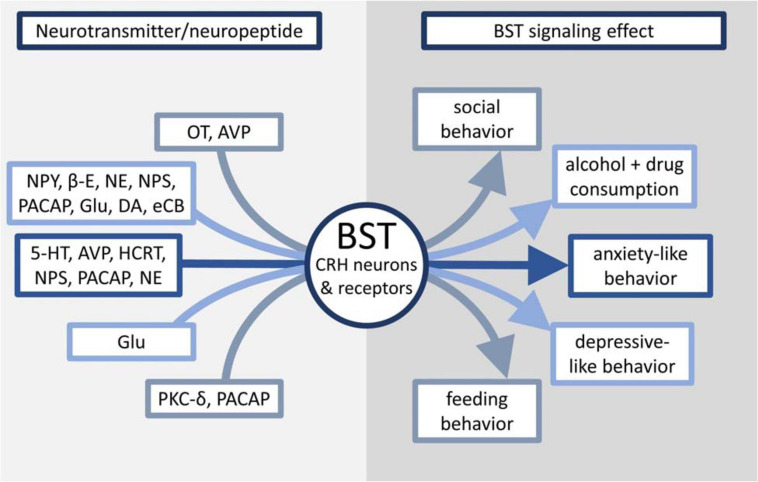
Overview of neurotransmitters linked to CRH signaling in the BST and their effects on maladaptive behaviors.

Because of the demonstrated impact of CRH on rodent behavior and its interaction with many other neurotransmitters and neuron types, this review will examine stress processing in the BST through the lens of CRH signaling and how it affects animal behavior, as well as possible implications of BST manipulation on the treatment of human mental health disorders. Figure 1 and Table 1 provide an overview of the effects discussed in this review.

**TABLE 1:** Summary of BST-CRH signaling studies.

Neurotransmitter/Neuropeptide	Target receptor	Target region	Treatment	Effect
5-HT	5-HT_1__A_	BSTd	Activation of 5-HT DRD projections	↓ Anxiety-like behavior (Garcia-Garcia et al., 2018)
	5-HT_2__c_	BST	Activation of 5-HT DRD projections	↑ Anxiety-like behavior (Marcinkiewcz et al., 2016)
OT	OTR	BSTam	OTR antagonist administration	↓ Stress-induced social avoidance in female mice (Duque-Wilckens et al., 2018)
AVP	V1aR	BSTmv	V1aR antagonist administration	↑ Sex and context–specific anxiety-like behavior (Duque-Wilckens et al., 2016)
	V1bR	Pituitary	Immobilization stress	↑ HPA axis activation (Dinan and Scott, 2005)
HCRT	—	BSTadl	BST-CRH activation of HCRT neurons	↑ Anxiety-like behavior and arousal (Giardino et al., 2018)
NPS	NPSR	—	i.c.v. NPS administration	↓ Anxiety-like behavior, ↑ arousal (Xu et al., 2004)
		lateral ventricle	CRHR1 antagonist and NPS administration	No increase in self-administered cocaine use (Pañeda et al., 2009)
PACAP	PAC_1_R	BSTov	Chronic variable mild stress paradigm	↑ Number of PACAP expressing cells in the BSTov (Hu et al., 2020)
		BST	i.c.v. PACAP infusion	↑ Anxiety-like behavior (Hammack et al., 2009a)
		BST	PAC_1_R antagonist administration	Blocked excessive alcohol consumption in dependent rats (Ferragud et al., 2020)
		BSTp	PACAP agonist administration	↑ Weight loss in rats (Kocho-Schellenberg et al., 2014)
PKC-∂	—	BSTov	Optogenetic activation of BSTov PKC-∂ neurons	↓ Feeding behavior (Wang et al., 2019)
Glutamate	NMDAR	BSTdl	Deletion of GluN2D subunit	↑ Depressive-like behavior (Salimando et al., 2020)
	AMPAR	BSTdl	Adolescent intermittent ethanol vapor exposure	↓ GluA2 subunit expression (Kasten et al., 2020)
NPY	Y1R	BST	Y1R activation	↓ Binge alcohol drinking (Pleil et al., 2015)
	Y2R	Lateral ventricle	Y2R antagonism	↓ Alcohol consumption (Thorsell et al., 2002)
β-E	μ and δ opioid receptors	BST	β-E deficient mice	↑ Alcohol consumption in female mice (Nentwig et al., 2019)
NE	α_2__a_-AR	BSTd	α_2__a_-AR agonist administration	↓ Anxiety-like behavior (Schweimer et al., 2005; Harris et al., 2018)
	β-AR	BST	NE administration	↑ CRH neuron activation (Snyder et al., 2019)
DA	D1R, D2R	BSTdl	DA administration	↑ glutamatergic transmission (Kash et al., 2008)
eCB	CB1R	BSTd	MAG lipase inhibitor administration	Normalized BST CRH neuron activation following alcohol withdrawal (Centanni et al., 2019)

*—Indicates value not specified by cited paper.*

## Bst Nomenclature and Anatomy

Papers vary in their usage of BST or BNST, as well as nucleus or nuclei in the BST name. Here, usage of the BST acronym and the word “nuclei” instead of “nucleus” in the BST name is to remain consistent with atlas nomenclature (Swanson, 2004; Lein et al., 2007; Shackman and Fox, 2016) and acknowledge the different functions of the BST nuclei. While the accepted number of BST nuclei varies (Gungor and Paré, 2016), this review will use the standard nomenclature system established by the Swanson (2004) rat brain atlas, which includes sixteen different regions, defined mostly by cytoarchitecture (Ju and Swanson, 1989). This is also the nomenclature used by the Allen Reference Atlases, with minor differences in hierarchical structure that include the removal of the cell sparse zone (BSTsz) and premedullary nucleus (BSTpm) as BST nuclei (Lein et al., 2007). Both the Swanson (2004) rat brain atlas and the Allen Mouse Brain Atlas (2004) divide the BST into two major subparts—the anterior and posterior divisions—with most research on stress and anxiety focusing on the anterior division (Kim et al., 2013). Important subdivisions for stress processing include the BSTam, oval nucleus BST (BSTov), dorsomedial nucleus BST (BSTdm), ventral nucleus BST (BSTv), and the anterolateral BST (BSTal) from the anterior division, and the dorsal BST (BSTd) from the posterior division.

**FIGURE 2:**
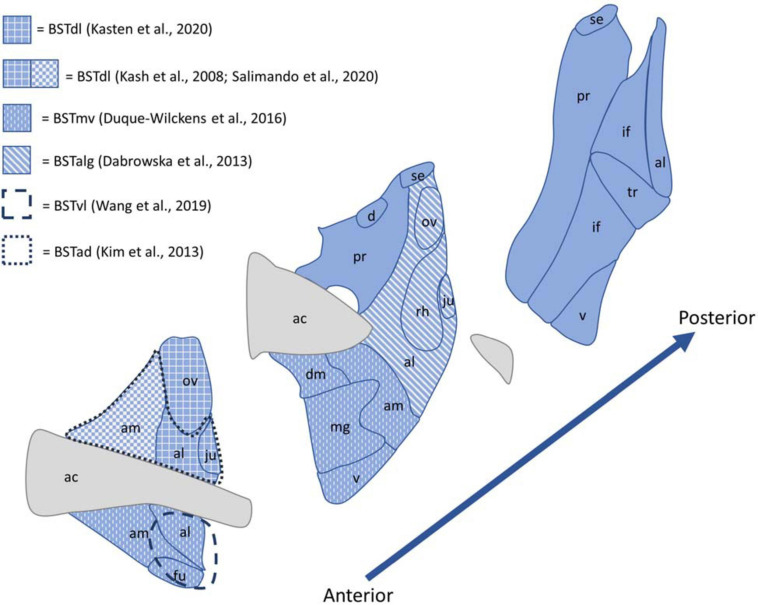
Diagram of the BST nuclei as described by the Allen Brain Adult Mouse Reference Atlas shown in blue, with white patterns and dashed lines indicating the nomenclature used to described the regions referenced by papers included in this review. Abbreviations: ac, anterior commissure; ad, anterodorsal; al, anterolateral area; alg, anterolateral cell group; am, anteromedial area; d, dorsal nucleus; dl, dorsolateral; fu, fusiform nucleus; if, interfascicular nucleus; ju, juxtacapsular nucleus; mg, magnocellular nucleus; mv, medioventral; ov, oval nucleus; pr, principal nucleus; rh, rhomboid nucleus; se, strial extension; tr, transverse nucleus; v, ventral nucleus.

Despite the existence of this standard nomenclature system, BST nomenclature is highly inconsistent across papers. This makes it difficult to compare data from paper to paper (Shackman and Fox, 2016), and because of the large number of nuclei in the BST (Gungor and Paré, 2016), accurate labeling and nomenclature is critical for reproducible and easily understood results. For example, if different papers call the anteromedial BST the anterodorsal, anteroventral, or dorsolateral BST, it is difficult for readers to determine if the papers focus on the same area. When discussing results from various papers, this review uses the same nuclei classification as used by the original authors. Figure 2 provides an illustration of the BST nuclei, as well as different nomenclatures used by the papers referenced in this review.

## Overview of CRH Signaling in the BST

Classically, CRH neurons are thought of in their role in hypothalamic-pituitary-adrenal (HPA) axis activation. In this role, there are at least two different populations of distinct CRH neurons—those in the BST and those in the paraventricular nucleus of the hypothalamus (PVH) (Dabrowska et al., 2013). The BST has been shown to contain mostly GABAergic neurons and a small amount of glutamatergic neurons (Moga and Saper, 1994). Similarly, CRH neurons in the BST are primarily GABAergic (Veinante et al., 1997), with Dabrowska et al. (2013) finding that 95% of CRH neurons in the BSTov colocalized with glutamic acid decarboxylase 67, a marker of GABAergic neurons, and had low colocalization with vesicular glutamate transporter 2, a marker of glutamatergic neurons. Conversely, PVH CRH neurons are primarily glutamatergic (Dabrowska et al., 2013), suggesting a unique role for each of the two populations in HPA axis activation. BST CRH neurons are concentrated in the BSTov (Ju et al., 1989), which is critical for the regulation of anxiety states (Kim et al., 2013) and may have a role as the master controller of the BST (Ch’ng et al., 2018).

Corticotropin releasing hormone signaling in the BST includes CRH neurons as well as the CRH receptors, which are widely distributed in the BST (Van Pett et al., 2000). CRH binding to CRH receptor type 1 (CRHR1) or CRH receptor type 2 (CRHR2) causes the receptor to couple to a G_sα_ protein, which then stimulates cAMP production through adenylate cyclase activation. cAMP activates protein kinase A (PKA), which phosphorylates downstream targets to carry out the effects of CRH signaling (for review, see Hillhouse and Grammatopoulos, 2006). Despite the prevalence of CRH neurons and receptors in the BST, in the anterolateral cell group of the BST (BSTalg), Dabrowska et al. (2013) found virtually no colocalization between CRH neurons and CRHR1 or CRHR2–expressing neurons.

CRH infusion to the BST was found to increase anxiety-like behavior in rats in the elevated plus maze (EPM) test, an effect mediated through CRHR1s (Sahuque et al., 2006). BST CRH infusion was also found to induce the reinstatement of cocaine-seeking behavior (Erb and Stewart, 1999), demonstrating the effect of CRH signaling on multiple forms of maladaptive behaviors. An important source of CRH in the BST comes from CRH projections from the CEA to the dorsolateral BST (BSTdl), and those projections are necessary for the induction of stress-induced anxiety (Pomrenze et al., 2019). In support of this conclusion, Ventura-Silva et al. (2020) found that CRH knockdown in the CEA reduced stress-induced anxiety-like behavior in rats and had no effect on the results of a fear-potentiated startle paradigm. This reinforces the role of CEA-BST circuit signaling in mediating chronic, and not acute, stress processing.

Hu et al. (2020) found that a chronic variable mild stress paradigm resulted in increased phosphorylated PKA in the anterior dorsolateral BST (BSTadl). Infusion of mice with a PKA-selective antagonist, H89, attenuated the anxiogenic effects caused by the stress paradigm, as measured by the EPM, OF, novelty suppressed feeding (NSF) tests. This suggests that PKA is necessary for transmitting anxiogenic signals caused by CRHR1 activation in the BSTov.

While many studies have linked BST activity and CRH signaling to anxiogenic activity, especially anxiogenic activity induced by CRHR1 activation (Bale and Vale, 2004), Henckens et al. (2017) found that CRHR2 activation is necessary for attenuating the stress response and promoting stress recovery. CRHR2 is highly expressed in the posterior BST (BSTp) (Van Pett et al., 2000), and optogenetic activation of CRHR2 neurons in the BSTp reduced anxiety-like behaviors in mice in the EPM and OF tests, while inhibition increased anxiety-like behaviors (Henckens et al., 2017). This contributes to the heterogeneity of the BST and also has implications for the treatment of PTSD and other stress-related disorders (Lebow and Chen, 2016; Henckens et al., 2017), especially considering that BST-CRHR2 levels can influence susceptibility to PTSD (Lebow et al., 2012).

As stated before, changes to BST CRH signaling have been implicated in many forms of maladaptive behaviors. Its effects are often contradictory (Lebow and Chen, 2016), potentially due to the different electrophysiological profiles of BST neurons, leading to anxiogenic or anxiolytic behaviors. Hammack et al. (2007) found three distinct populations of neurons in the BSTalg, Type I-III, defined by the neuronal electrophysiological response. Moreover, all three types have the potential to contain CRH and express different neurotransmitters and receptors, resulting in CRH neurons that fire under different neurochemical conditions (Dabrowska et al., 2013). This review will examine individually the effects of different neurotransmitters on CRH signaling in the BST and associated disorders.

## Serotonin

Serotonin (5-hydroxytryptamine; 5-HT) plays a major role in many psychiatric disorders and is a common treatment target in the form of selective serotonin reuptake inhibitors (SSRIs) (Craske et al., 2017). Its effects are mediated through seven families of 5-HT receptors, 5-HT_1__–__7_. Especially important for anxiety processing are the G_i/o_ protein–coupled 5-HT_1_ and G_q_ protein–coupled 5-HT_2_ receptor families (for review, see Hammack et al., 2009b). The BST, specifically the BSTdl and BSTv, receive 5-HT projections from the dorsomedial dorsal raphe nucleus (DRD) (Commons et al., 2003). Levita et al. (2004) found that activation of the BSTal by DRD 5-HT inputs caused hyperpolarization and inhibition with anxiolytic effects, measured by the acoustic startle reflex. DRD 5-HT neurons are activated by CRH (Lowry et al., 2000), and because of the high density of CRH in the BST, it was questioned if 5-HT and CRH signaling between the DRD and BST formed a feedback loop.

In support of this, Donner et al. (2018) found that rat Tph2, the rate-limiting enzyme of 5-HT synthesis, and *tph2* mRNA expression in the DRD were increased after stress exposure. Infusion of the BST with urocortin-1 (Ucn-1), a CRHR1 and CRHR2 agonist (Vaughan et al., 1995), was shown by Donner et al. (2020) to increase DRD *tph2* mRNA, suggesting that DRD serotonergic neurons are regulated by BST-CRH neurons and help promote anxiety-like states. Optogenetic activation of 5-HT projections from the DRD to the BSTd resulted in reduced c-fos expression and anxiolytic effects on behavior in the EPM, OF, and NSF tests, while inhibition had anxiogenic effects, mediated through 5-H_1__A_ receptors in the BSTd (Garcia-Garcia et al., 2018).

Marcinkiewcz et al. (2016) found that activation of DRD projections to the BST, operating through 5-HT_2__C_ receptors, was anxiogenic in the EPM and NSF tests and silenced anxiolytic BST outputs to the ventral tegmental area (VTA) and lateral hypothalamus (LH). The different 5-HT receptor families might explain the opposing effects found by Donner et al. (2020) and Garcia-Garcia et al. (2018). However, a knockdown model of the 5-HT_1__A_ receptor yielded no effect on anxiety-like behavior, suggesting that without activation, the receptor may not be in great enough abundance to yield a physiologically relevant effect on behavior (Marcinkiewcz et al., 2019). More research is therefore necessary to further elucidate the role of 5-HT_1__A_ receptors in regulating anxiety-like behavior, especially considering the implications of the 5-HT_1__A_ receptor on SSRI treatment success (Quentin et al., 2018).

## Oxytocin

Oxytocin (OT), beyond acting as a hormone, plays a role in the regulation of anxiety processing in the central nervous system and has been the subject of several thorough reviews (Janeček and Dabrowska, 2019; Steinman et al., 2019). OT is primary synthesized in the PVH and supraoptic nuclei of the hypothalamus, and while some of those neurons project to the BST and act as a source of OT, OT is also synthesized in the BST itself (Lee et al., 2009). Intraperitoneal OT administration has been found to have anxiolytic effects in rats, measured by the emergence and social interaction (SI) tests, as well as decrease alcohol consumption (Bowen et al., 2011). The G_q_ protein–coupled OT receptors (OTRs) in the BSTam have also been linked to social avoidance in female, but not male, mice, again highlighting the sexual dimorphism of the BST (Duque-Wilckens et al., 2018). More specifically, Dabrowska et al. (2011) found that OT-immunoreactive (ir) fibers in the BSTov colocalized with CRHR2-ir fibers and had perisomatic contact with CRH-ir neurons. These neurons project to the magnocellular portion of the PVH (Dong et al., 2001), forming a reciprocal feedback loop to regulate activation of the HPA axis (Dabrowska et al., 2011).

## Arginine Vasopressin

Like OT, changes to arginine vasopressin (AVP) signaling in the medioventral BST (BSTmv), including expression of AVP and its receptor, the G_q_ protein–coupled vasopressin V1a receptor (V1aR), have been implicated in sex-specific responses to social defeat and stress (Duque-Wilckens et al., 2016). The BST is a major extrahypothalamic source of AVP, and BST AVP neurons project to many forebrain and midbrain structures (Leeuwen and Caffé, 1983; de Vries and Miller, 1999). Administration of a V1aR antagonist to the BSTmv had anxiogenic effects in both male and female mice in the SI test, but in female mice, the effect only occurred in social contexts (Duque-Wilckens et al., 2016). AVP works with CRH to regulate the HPA axis (Kormos and Gaszner, 2013), and sustained AVP levels due to chronic stress, operating through G_q_ protein–coupled V1b receptors (V1bRs; also known as V3R), lead to increased HPA activation through the stimulation of adrenocorticotropin (ACTH) release (Dinan and Scott, 2005). Given the role of CRH neurons in HPA axis activation and the presence of V1bRs in the BST (Hernando et al., 2001), it is possible that BST AVP signaling, mediated through CRH signaling, is involved in multiple pathways, including the processing of anxiogenic stimuli and HPA axis activation. Clinically, elevated AVP levels have been seen in depressed patients, and V1bR antagonists are being investigated in preclinical models for their antidepressant and anxiolytic effects (for review, see Alldredge, 2010).

## Hypocretin

Hypocretin (HCRT; orexin) is expressed in a subset of LH neurons and is implicated in the regulation of emotional behavior (Soya and Sakurai, 2020). HCRT-LH neurons receive inputs from CRH-BST neurons, and HCRT activation via CRH-BST neurons is associated with negative valence and behavioral avoidance in the real-time place test (Giardino et al., 2018). HCRT is closely associated with wakefulness and narcolepsy (Soya and Sakurai, 2020) because of its role in the regulation of vigilance and arousal states (Boutrel and de Lecea, 2008), so it raises the question of if the BST-HCRT-LH pathway can help explain the sleep disruptions commonly associated with anxiety (American Psychiatric Association, 2013). It is possible that upregulation of CRH neurons in the BST and subsequent activation of HCRT-LH neurons may therefore contribute to the increased arousal states associated with anxiety and other altered mood states, as well as contribute to the role of the BST in regulating addiction (Snyder et al., 2019).

## Neuropeptide S

Neuropeptide S (NPS) and its receptor, the G_s_/G_q_ protein–coupled NPS receptor (NPSR; also known as GPR154), make up a deorphanized GPCR system implicated in the regulation of anxiety, arousal, and wakefulness (Xu et al., 2004), as well as drug and alcohol–related behaviors (Cannella et al., 2009; Pañeda et al., 2009). While NPS is expressed in only a few regions of the rat brain, notably in a group of neurons between the locus coeruleus and Barrington’s nucleus (Xu et al., 2007), the NPSR is more widely distributed and is found in the BSTv (Leonard and Ring, 2011). Administration of NPS in rodents resulted in increased arousal, determined by locomotor activity, and decreased anxiety-like behavior, determined by the EPM, OF, and light-dark box tests (Xu et al., 2004), similar to the effect of nicotine (Koob and Greenwell, 2004). Pañeda et al. (2009) found that in mice, i.c.v. administration of NPS to the lateral ventricle induced cocaine-seeking behavior in a CRHR1-dependent manner. When they either administered the CRHR1 antagonist antalarmin or used a CRHR1 knockout model, NPS administration failed to increase self-administered cocaine use. Following morphine withdrawal, NPSR transcript prevalence decreased in the BST, and NPS administration decreased anxiety-like behavior associated with withdrawal (Ghazal et al., 2013). NPS has also been linked to LH-HCRT neurons (Cannella et al., 2009), further linking the BST and CRH signaling to addiction pathways as discussed in the previous HCRT section.

## Pituitary Adenylate Cyclase–Activating Polypeptide

Pituitary adenylate cyclase–activating polypeptide (PACAP), operating through the G_s_/G_q_ protein–coupled PAC_1_ receptor (PAC_1_R) (Pisegna and Wank, 1996), is an upstream regulator of CRH that innervates CRH neurons in the BSTov, where it contributes to increased anxiety states and can activate the HPA axis (for review, see Lebow and Chen, 2016). Major sources of PACAP to the BST include the PVH and dorsal vagal complex (Kozicz et al., 1998). Hu et al. (2020) found that subjecting male mice to a chronic variable mild stress paradigm significantly increased the number of PACAP expressing cells in the BSTov, as well as *Pacap* mRNA expression in the BSTadl. Effects on anxiety-like behavior in male rats following PACAP infusion into the BST, measured by the light-enhanced startle test, are long lasting, occurring for at least 1 week post infusion (Hammack et al., 2009a). The anterodorsal BST (BSTad) had been previously shown to have anxiolytic effects (Kim et al., 2013), highlighting the heterogeneity and complexity of the BST.

While classically PACAP has been thought of in terms of its regulation of anxiety-like behavior, recently it has also been implicated in the regulation of alcohol consumption. Ferragud et al. (2020) demonstrated that exposure of rats to chronic intermittent ethanol vapors increased PACAP levels in the BST, and that administration of a PAC_1_R antagonist to the BST blocked excessive alcohol consumption in ethanol-dependent rats. This research highlights the diverse natures of neurotransmitters in the BST and how they may have multifaceted roles in behavioral regulation.

Additionally, PACAP has been implicated in feeding regulation, with Kocho-Schellenberg et al. (2014) finding that infusion of the BSTp with a PACAP agonist resulted in a dose-dependent weight loss for the 24 h following the infusion in both male and female rats.

Susceptibility to the anxiogenic effects of PACAP depends on previous stress exposure, as well as sex (King et al., 2017). Female rats did not display the same increase in startle reflex as male rats when infused with PACAP in the BST following chronic stress exposure (King et al., 2017). More research is needed to investigate the effects of PACAP expression in female rodents. This is especially important considering that PACAP has been implicated as a potential treatment target for PTSD (Lebow and Chen, 2016), and that women are more than twice as likely as men to develop PTSD following a traumatic event (King et al., 2017).

## Protein Kinase C Delta

The BSTov contains protein kinase C delta (PKC-∂) neurons, which help mediate feeding behaviors through a novel circuit proposed by Wang et al. (2019). The PKC family is composed of ten different isoforms that are sorted into three subfamilies, with PKC-∂ belonging to the novel PKC subfamily (Sanchez-Bautista and Nicolas, 2013). PKC-∂ is activated when CRH binds to the CRHR1 or CRHR2 receptor, which can then couple to a G_q_ protein and activate phospholipase C (PLC). PLC then catalyzes the production of inositol trisphosphate (IP3) and diacylglycerol (DAG), which activates PKC-∂. Activating BSTov PKC-∂ neurons suppressed feeding in mice, while silencing those neurons increased feeding (Wang et al., 2019). BSTov PKC-∂ neurons innervate and inhibit ventrolateral BST (BSTvl) neurons, demonstrating the interconnectivity of the BST subnuclei (Wang et al., 2019). Those BSTvl neurons then project to the LH to promote feeding behaviors, which explains how activating BSTov PKC-∂ neurons suppresses feeding by inhibiting the BSTvl neurons that are necessary to activate the LH neurons (Wang et al., 2019). PKC-∂ and CRH signaling may therefore link anxiety-like and feeding behaviors—in support of this conclusion, Hu et al. (2020) found that chronically stressed mice had decreased weight gain compared to controls.

## Glutamate

Glutamate operates though receptors that can be classified as either ionotropic, composed of *N*-methyl-D-aspartate receptors (NMDARs) and α-amino-3-hydroxy-5-methyl-4-isoxazolepropionic acid receptors (AMPARs), or metabotropic, and BST neurons contain both (Gracy and Pickel, 1995; Grueter and Winder, 2005; Grueter et al., 2006). Specifically, BST CRH neurons express NMDARs containing the GluN2D subunit (Salimando et al., 2020). NMDARs have been previously implicated in mental health disorders and their treatments (Ghasemi et al., 2014), and consistent with that, Salimando et al. (2020) found that deletion of the BST-GluN2D subunit led to increased depressive-like behaviors in mice as measured by the FS test, potentially because of decreased BST excitatory synaptic potentiation and subsequent increased excitatory drive onto BST-CRH neurons. There was no change in anxiety-like behavior, measured by the OF and elevated zero maze tests. GluN2D receptors were found to be localized in the BST to the BSTdl (Salimando et al., 2020), possibly contributing to the role of the BSTd in negative affect and valence processing (Kim et al., 2013). This research demonstrates the role of the BST in regulating emotional behavior beyond chronic fear responses and connects it to the regulation of mood disorders, such as major depressive disorder (MDD). Showing the translational potential between pre-clinical models and human studies, NMDAR antagonists are being investigated in clinical trials for the treatment of MDD and bipolar disorder (Ghasemi et al., 2014), and in 2019, the United States Food and Drug Administration (FDA) approved Spravato (esketamine), a NMDAR antagonist, in a nasal spray format for use in conjunction with antidepressants for the treatment of treatment-resistant depression (Daly et al., 2019; Popova et al., 2019; US Food and Drug Administration, 2019). However, concerns have been raised about the treatment length and long-term effects of intranasal esketamine treatment (Schatzberg, 2019; Zheng et al., 2020), and additional clinical trials will be key for improving MDD treatment using esketamine.

Bed nuclei of the stria terminalis neurons also contain AMPARs, a type of excitatory ionotropic glutamate receptor made up of four different subunits, GluA1-GluA4 (for review, see Chater and Goda, 2014). Kasten et al. (2020) found that adolescent intermittent ethanol vapor exposure (AIE) reduced the GluA2 AMPAR subunit expression in the BSTdl. They also found that the mGluR1 antagonist DHPG decreased long term depression in female, but not male, mice treated with AIE. CRHR1 activation resulting in PKA activation in the BST has been shown to cause the phosphorylation of GluR1 AMPAR subunits (Hu et al., 2020), so while the glutamate receptor research discussed above was not directly linked to CRH neurons, considering the previous connections made between CRH neurons in the BST with both AUDs and MDD, it is possible that CRH neurons influence this phenomenon and remains an area for future exploration.

## Neuropeptide Y

Neuropeptide Y (NPY) signaling has been implicated in alcohol abuse disorders (Pleil et al., 2015), feeding regulation (Higuchi, 2012), and fear memory (Bartsch et al., 2020). The majority of NPY input to the BST comes to the BSTa from agouti-related protein (AgRP) neurons in the arcuate nucleus (ARC) that co-release NPY, although the BST also contains NPY interneurons (Betley et al., 2013; Kash et al., 2015). The G_i/o_ protein–coupled NPY receptor (NPYR) family has five receptors, although only four are active in humans, and NPYR activation frequently results in inhibition of adenylyl cyclase and cAMP accumulation (Pedragosa Badia et al., 2013). Since CRH causes cAMP production, NPY and CRH often have opposing effects on behavior. In fact, while CRH signaling in the BST promotes alcohol seeking–behaviors (Nentwig et al., 2019), Pleil et al. (2015) found that activation of the Y1 NPY receptor (Y1R) reduced binge alcohol drinking in mice without modifying anxiety-like behavior in the OF test. They demonstrated that Y1R activation inhibited PKA activity and increased the frequency of inhibitory postsynaptic currents in BST CRH neurons, suggesting that Y1R activation results in the inhibition of CRH signaling. However, signaling through the Y2 NPY receptor (Y2R), which is also coupled to a G_q_ protein (Misra et al., 2004), has been demonstrated to have the opposite effect, with Thorsell et al. (2002) finding that administration of a Y2R antagonist to the lateral ventricle in rats decreased alcohol consumption. For further discussion about NPY’s role in alcohol consumption, see Ch’ng et al. (2018).

## β-Endorphin

β-endorphin (β-E) has been demonstrated to have a role in AUDs and binge drinking behaviors, and targets μ-opioid receptors in the VTA or κ-opioid receptors in the NAc (Herz, 1997). Alcohol (EtOH) activates these receptors, which are part of the dopaminergic reward system, potentially starting an addiction cycle (Herz, 1997). Specifically, the BST has been linked to the withdrawal and relapse phases of addiction (Avery et al., 2016). Like CRH, β-E can regulate the HPA axis. CRH upregulates the release of β-E, which binds to G_i_ protein–coupled μ- and δ-opioid receptors to inhibit the release of CRH in the PVH through a negative feedback loop (for review, see Al-Hasani and Bruchas, 2011; Crews et al., 2015). Nentwig et al. (2019) found sex-specific differences in the effects of β-E on binge drinking behavior, with β-E–deficient female, but not male, mice consuming more alcohol than both β-E–deficient male mice and control female mice. β-E–deficient mice were found to have greater basal *Crh* expression in the BST, which is reduced upon alcohol consumption to control levels. Increased alcohol consumption in mice with increased *Crh* expression suggests that the behavior is driven, at least partially, by a desire to reduce stress (Nentwig et al., 2019).

## Norepinephrine

Norepinephrine (NE), acting through α- and β-adrenergic receptors (ARs), plays a key role in BST CRH neuron regulation. The main sources of NE input to the BST primarily innervate the BSTv and come from the A1, A2, and A5 brainstem nuclei (Crestani et al., 2013). In the AR family of GPCRs, there are three different types—α_1_ coupled to G_q_ proteins, α_2_ coupled to G_i/o_ proteins, and β coupled to G_s_ proteins—with each type being further divided into three subtypes (Bylund, 2013). Specifically, administering α_2__a_-ARs agonists to the BST have been shown to reduce anxiety-like responses in rats in fear-potentiated and light-enhanced startle tests (Schweimer et al., 2005) through inhibiting NE signaling (Buffalari et al., 2012). Harris et al. (2018) found this effect to be specific to the BSTd. α_2__a_-AR agonists administered during restraint stress decreased CRH activation in the BST, with sex-specific effects (Fetterly et al., 2019). While α-ARs mediate glutamatergic inputs into BST CRH neurons in an inhibitory manner (Fetterly et al., 2019), β-ARs have been shown to depolarize BST CRH neurons when treated with NE, leading to greater neuronal activation (Snyder et al., 2019). β-AR activation occurs during both stress and EtOH administration, further tying the BST to AUDs (Snyder et al., 2019). Snyder et al. (2019) then go on to propose that NE can alter BST CRH neuron excitability through two distinct pathways: one causing excitatory signaling through β-ARs that is sensitive to stress and EtOH exposure, and one causing inhibitory signaling through α-ARs that is not. This directly opposes findings from Fetterly et al. (2019) that administration of an α_2__a_-AR agonist decreased BST CRH cFos expression during restraint stress, and considering the large overlap of authors between the two papers, this suggests a high level of variability in these stress assays and the need to further delineate the distinct roles of different AR types in response to stress.

## Dopamine

Dopamine (DA) signaling in the BST has been linked to dysregulated CRH signaling and reinstatement of drug-seeking behavior (Silberman and Winder, 2013). Many drugs have been found to increase basal DA levels in the BST (Carboni et al., 2000). Main sources of DA to the BST, and in particular the BSTdl, include the VTA, dorsal raphe nucleus, and periaqueductal area (Park et al., 2012). Kash et al. (2008) found that DA application to the BSTdl operated through D1 and D2-like receptors, and enhanced glutamatergic transmission of BST neurons in a CRHR1-dependent manner. More specifically, based on the location of D1 mRNA, DA release may upregulate local CRH release in the BSTov (Daniel and Rainnie, 2016). This demonstrates the importance of BST CRH neuron activation in areas beyond the regulation of anxiety-like behavior, and CRHR1 antagonists remain a potential avenue for the development of therapeutics that help prevent the reinstatement of drug-seeking behavior.

## Endocannabinoids

Endocannabinoids (eCBs), primarily 2-arachadonylglycerol (2-AG) and *N*-arachidonylethanolamine (AEA), are lipid ligands that signal through two different types of G_i/o_ protein–coupled cannabinoid receptors (CBRs), CB1R and CB2R, to help regulate the stress response (for review, see Morena et al., 2016). CB1Rs are widely distributed throughout the brain, and are highly expressed in the BSTd (Puente et al., 2010). Centanni et al. (2019) found greater activation of BSTd CRH neurons following alcohol abstinence in female mice. Treatment with a monoacylglycerol (MAG) lipase inhibitor, which enhances endogenous 2-AG levels, prevented the increase in neuronal activation in the BSTd, specifically through an insular-BSTd circuit (Centanni et al., 2019). Because of the importance of BSTd signaling in the regulation of maladaptive behaviors (Lebow and Chen, 2016), targeting CB1R receptors in that region could comprise a new therapeutic target for the treatment of human mental health disorders (Centanni et al., 2019). However, current research into the link between CRH neuron activation and eCBs in the BST is limited and remains an ongoing area of study.

## Therapeutic and Translational Potential

### Current Applications in Deep Brain Stimulation Therapy in Humans

The role of the BST in sustained fear processing is not limited to rodents. Heightened levels of BST activity in humans have been demonstrated in patients with general anxiety disorder, PTSD, panic disorder, and specific phobias (for review, see Knight and Depue, 2019; Awasthi et al., 2020). While more research into the human BST is needed, there has already been success in treating certain disorders using deep brain stimulation (DBS) to the BST. Luyten et al. (2016) demonstrated in a double-blind crossover study that DBS using electrodes implanted in the BST caused a significant reduction in obsessive-compulsive disorder (OCD) symptoms. While the BST has traditionally been considered outside the scope of OCD neurocircuitry, the success in DBS treatment suggests adaptations to the current model are needed (Luyten, 2020).

Bed nuclei of the stria terminalis DBS was also used to treat a patient with severe MDD and comorbid anorexia nervosa, resulting in significantly reduced scores on depression indices and a reduction in anxiety concerning food and eating behaviors (Blomstedt et al., 2017). In another study, Raymaekers et al. (2017) also found positive effects for the treatment of MDD using BST stimulation. However, sample sizes were limited and more research is needed to more definitely conclude the efficacy of BST DBS for MDD treatment.

### Clinical Trials of CRHR1 Antagonists

Based on the plethora of preclinical evidence implicating the CRH signaling pathway in maladaptive behaviors, several clinical studies have investigated CRHR1 antagonists as potential therapeutic targets for the treatment of anxiety, PTSD, and AUDs. However, they had limited success. Two studies have examined the effects of CRHR1 antagonist verucerfont (formerly GSK561679) on alcohol craving (Schwandt et al., 2016) and PTSD (Dunlop et al., 2017), with both studies finding little effect. However, a study of the CRHR1 antagonist pexacerfont found that the effect of the drug on reducing food cravings was worth further study—although it should be noted that this study was stopped early for legal reasons unrelated to the study safety or outcome (Epstein et al., 2016). While these results might suggest reduced translational potential of CRHR1 antagonists for the treatment of anxiety and other mental health disorders, it is important to consider that the drug administration in humans was not specific to a particular brain region, which is a notable difference from preclinical models. Because injecting drugs directly into the human BST is limited in feasibility due to patient burden and the small size of the BST (Knight and Depue, 2019), it is critical to develop CRHR1 antagonists that are specific to the systems they are targeting. Another limitation of clinical trials is that the experimental design might not exactly match what was being tested in the preclinical model, such as the phase of the addiction model in AUDs (Pomrenze et al., 2017). Careful recruiting of patients to match the conditions of the preclinical model as closely as possible may yield better results. In addition, there could be other factors in human neurobiology that influence CRHR1 antagonist effects that are not found in preclinical models. That is why Grillon et al. (2019) recommend using a combination of preclinical models and experimental psychopathology using healthy human volunteers to aid the process of drug discovery and validation, going so far as to recommend adding a new testing criteria between phase I and phase II of clinical trials based on anxiety-potentiated startle (Grillon and Ernst, 2020).

## Conclusion and Future Directions

The BST serves as a hub for the regulation of chronic fear responses and other behaviors that can lead to anxiety and mood disorders. The BSTp has mostly anxiolytic effects, largely mediated through CRHR2s. In contrast, the BSTa, particularly the BSTov, has been shown to mediate anxiety-like behavior and is also implicated in mood disorders and drug and alcohol use. In the BSTa, changes to CRH signaling are critical for these effects. Activation by a wide variety of neurotransmitters and receptors were all demonstrated to affect maladaptive behavior in pre-clinical models. Due to the complicated nature of CRH interaction with these neurotransmitters, careful experimental design using standardized testing procedures and nuclei classifications will aid in the interpretation of results and enhance reproducibility across labs. Because there are so many neurotransmitters and receptors that are connected to CRH signaling, targeting CRH and the CRHRs presents a potential avenue to cover a broad array of pathways in the treatment of anxiety, mood, and substance use disorders. Considering the high impact those disorders have on society and the economy, and the demonstrated impact of BST treatment on some of these disorders, further research is needed to advance the understanding of how changes in CRH signaling impacts these disorders. In particular, considering the sexually dimorphic nature of the BST, and the gender bias in pre-clinical research (Ciccocioppo, 2017), more research is needed involving both male and female rodents to help elucidate sex-specific differences in maladaptive behaviors. While previous clinical trials for CRHR1 antagonists have not been successful for the treatment of cravings, given the complexity of BST CRH signaling, there is still room to investigate its role in other disorders, as well as treatments targeting different receptors or pathways linked to CRH signaling.

## Author Contributions

CEY wrote and revised the manuscript. QT provided critical feedback regarding content and structure of the manuscript. Both authors contributed to the article and approved the submitted version.

## Conflict of Interest

The authors declare that the research was conducted in the absence of any commercial or financial relationships that could be construed as a potential conflict of interest.
